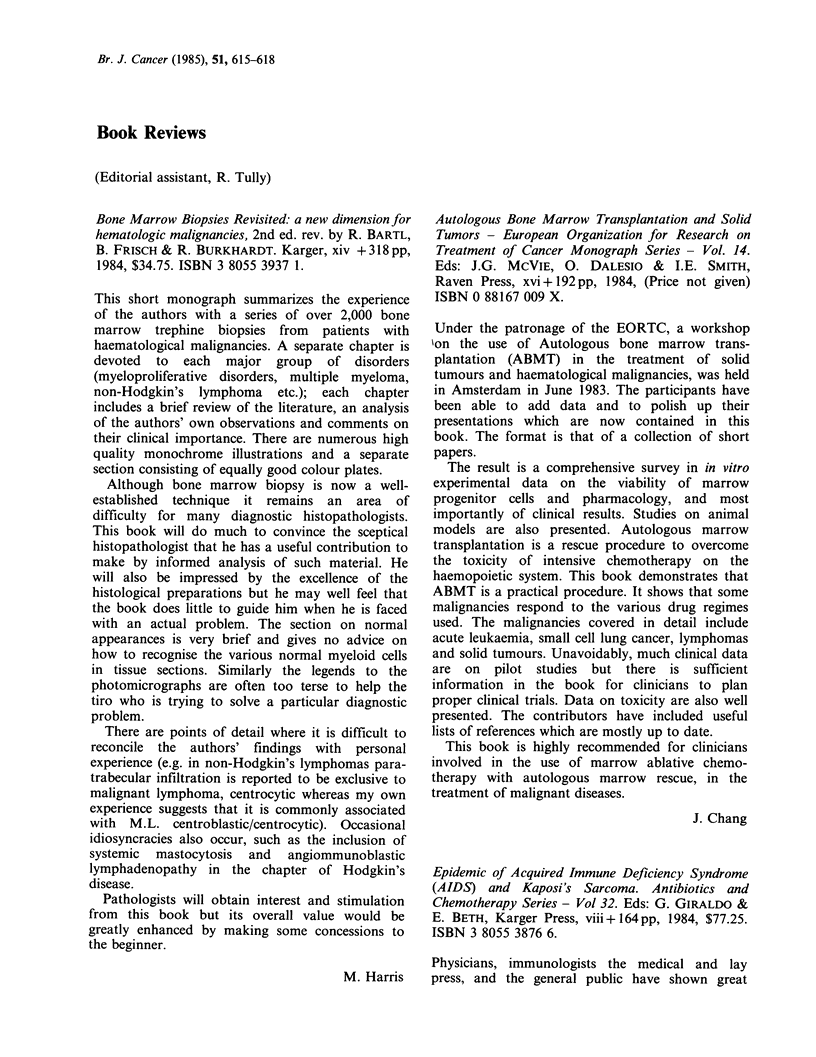# Bone Marrow Biopsies Revisited: a new dimension for hematologic malignancies

**Published:** 1985-04

**Authors:** M. Harris


*Br. J. Cancer* (1985), **51,** 615–618

### Book Reviews

(Editorial assistant, R. Tully)

*Bone Marrow Biopsies Revisited: a new dimension for hematologic malignancies,* 2nd ed. rev. by R. BARTL, B. FRISCH & R. BURKHARDT. Karger, xiv +318 pp, 1984, $34.75. ISBN 3 8055 3937 1.

This short monograph summarizes the experience of the authors with a series of over 2,000 bone marrow trephine biopsies from patients with haematological malignancies. A separate chapter is devoted to each major group of disorders (myeloproliferative disorders, multiple myeloma, non-Hodgkin's lymphoma etc.); each chapter includes a brief review of the literature, an analysis of the authors' own observations and comments on their clinical importance. There are numerous high quality monochrome illustrations and a separate section consisting of equally good colour plates.

Although bone marrow biopsy is now a well-established technique it remains an area of difficulty for many diagnostic histopathologists. This book will do much to convince the sceptical histopathologist that he has a useful contribution to make by informed analysis of such material. He will also be impressed by the excellence of the histological preparations but he may well feel that the book does little to guide him when he is faced with an actual problem. The section on normal appearances is very brief and gives no advice on how to recognise the various normal myeloid cells in tissue sections. Similarly the legends to the photomicrographs are often too terse to help the tiro who is trying to solve a particular diagnostic problem.

There are points of detail where it is difficult to reconcile the authors' findings with personal experience (e.g. in non-Hodgkin's lymphomas para-trabecular infiltration is reported to be exclusive to malignant lymphoma, centrocytic whereas my own experience suggests that it is commonly associated with M.L. centroblastic/centrocytic). Occasional idiosyncracies also occur, such as the inclusion of systemic mastocytosis and angiommunoblastic lymphadenopathy in the chapter of Hodgkin's disease.

Pathologists will obtain interest and stimulation from this book but its overall value would be greatly enhanced by making some concessions to the beginner.

M. Harris